# Increased cerebrospinal fluid adenosine 5'-triphosphate in patients with amyotrophic lateral sclerosis

**DOI:** 10.1186/s12883-021-02288-4

**Published:** 2021-06-30

**Authors:** Takamasa Nukui, Atsushi Matsui, Hideki Niimi, Tomoyuki Sugimoto, Tomohiro Hayashi, Nobuhiro Dougu, Hirofumi Konishi, Mamoru Yamamoto, Ryoko Anada, Noriyuki Matsuda, Isao Kitajima, Yuji Nakatsuji

**Affiliations:** 1 Department of Neurology, Faculty of Medicine, University of Toyama, 2630 Sugitani, Toyama, 930-0194 Japan; 2 Department of Clinical Laboratory and Molecular Pathology, Graduate School of Medicine and Pharmaceutical Science for Research, University of Toyama, 2630 Sugitani, Toyama, 930-0194 Japan; 3 Faculty of Data Science, Graduate School of Data Science, University of Shiga, 1-1-1 Banba Hikone, Shiga, 522-8522 Japan

**Keywords:** ALS, Cerebrospinal fluid, ATP, Biomarker

## Abstract

**Background:**

Extracellular adenosine 5'-triphosphate (ATP) has been suggested to cause neuroinflammation and motor neuron degeneration by activating microglia and astrocytes in amyotrophic lateral sclerosis (ALS). Since we have developed a highly sensitive ATP assay system, we examined cerebrospinal fluid (CSF) ATP levels in patients with ALS whether it can be a useful biomarker in ALS.

**Methods:**

Forty-eight CSF samples from 44 patients with ALS were assayed for ATP with a newly established, highly sensitive assay system using luciferase luminous reaction. CSF samples from patients with idiopathic normal pressure hydrocephalus (iNPH) were assayed as a control. Patients were divided into two groups depending on their disease severity, as evaluated using the Medical Research Council (MRC) sum score. Correlations between the CSF ATP levels and other factors, including clinical data and serum creatinine levels, were evaluated.

**Results:**

CSF ATP levels were significantly higher in patients with ALS than in the iNPH (716 ± 411 vs. 3635 ± 5465 pmol/L, *p *< 0.01). CSF ATP levels were significantly higher in the more severe group than in the iNPH group (6860 ± 8312 vs. 716 ± 411 pmol/L, *p *< 0.05) and mild group (6860 ± 8312 vs. 2676 ± 3959 pmol/L, *p *< 0.05) respectively. ALS functional rating scale-revised (ALSFRS-R) (37.9 ± 5.7 vs. 42.4 ± 2.8, *p *< 0.01) and serum creatinine levels (0.51 ± 0.13 vs. 0.68 ± 0.23 mg/dL, *p *< 0.05) were significantly lower in the severe group than in the mild group respectively. A negative correlation of CSF ATP levels with MRC sum score was demonstrated in the correlation analysis adjusted for age and sex (r = -0.3, *p *= 0.08).

**Conclusions:**

Extracellular ATP is particularly increased in the CSF of patients with advanced ALS. CSF ATP levels may be a useful biomarker for evaluating disease severity in patients with ALS.

## Introduction

Amyotrophic lateral sclerosis (ALS) is a progressive neurodegenerative disease characterized by degeneration of motor neurons in the motor cortex, brain stem, and spinal cord, which leads to muscle atrophy, paralysis, respiratory failure, and death within several years of disease onset [1]. Although the pathogenesis of ALS remains to be fully elucidated, aggregation of cytoplasmic proteins, such as nuclear TAR DNA-binding protein 43 (TDP-43) [2], and neuroinflammation induced by activated microglia and astrocytes may be attributed to motor neuron death [3]. Several blood or cerebrospinal fluid (CSF) proteins and cytokines related to muscle atrophy and motor neuron death have been used as biomarkers for ALS. For example, plasma creatinine has been suggested as a surrogate marker in ALS because it is related to muscle mass of patients and is predictive of the patient’s muscle strength or mortality risk [4, 5]. Patients with ALS exhibit lower serum uric acid levels than healthy controls, and this decreased uric acid level correlates to disease progression [6]. Recently, neurofilament light chain (NfL) was reported to be increased in CSF in patients with ALS compared with other neurological diseases, and is suggested to be not only a diagnostic biomarker but also a predictor of disease progression [7].

Adenosine 5'-triphosphate (ATP) is an energy source in living cells generated in mitochondria. In early disease stage of ALS, mitochondrial dysfunction cause motor neuron death by disrupting intracellular calcium homeostasis, and by increasing production of reactive oxygen species (ROS) [8]. ATP is also acts as a signaling molecule in the nervous system by binding to its specific receptor [9]. Many studies have suggested that extracellular ATP is involved in the pathophysiology of several neurological diseases such as Alzheimer’s disease [10, 11], Parkinson’s disease [12, 13], multiple sclerosis [14, 15], and ALS [16]. In vitro experiments have shown that a high concentration of extracellular ATP activates the microglia and astrocytes, promotes T cell infiltration, and induces neurotoxic inflammation accompanied by the degeneration of motor neurons [17]. Therefore, CSF ATP levels may change in accordance with disease severity in patients with ALS. We have recently established a highly sensitive assay for ATP using a luciferase luminous reaction and reported that ATP levels reflect disease severity and the efficacy of treatment in patients with mitochondrial myopathy, encephalopathy, lactic acidosis, and stroke like episodes (MELAS) [18]. In this report, we assayed ATP levels in the CSF of patients with ALS to investigate whether it is a useful biomarker for disease severity or other clinical factors in ALS.

## Materials and methods

### Patients and ethics

**Fig. 1:**
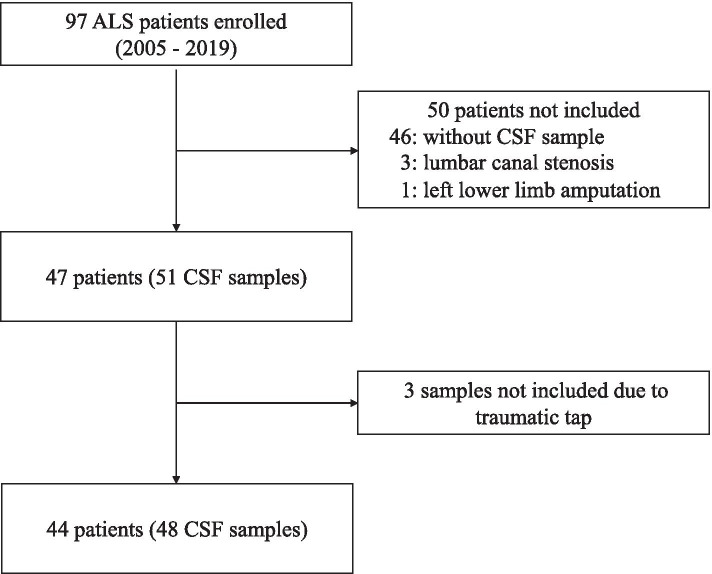
Flowchart of cerebrospinal fluid sample selection. ALS; amyotrophic lateral sclerosis, CSF; cerebrospinal fluid

Ninety-seven patients with ALS admitted to Toyama University Hospital between 2005 and 2019 were enrolled. The diagnosis of possible, probable, or definite ALS was made according to the Awaji criteria [19]. Among the 97 patients, 46 without cerebrospinal fluid (CSF) samples were excluded. Three patients with neuropathic pain due to lumbar canal stenosis were also excluded because CSF ATP levels may be upregulated in such patients [20]. One patient who had lost her leg was excluded due to the impossibility of evaluating the Medical Research Council (MRC) sum score. The MRC sum score was defined as the sum of MRC scores from six muscles in the upper (shoulder abduction, elbow flexion, wrist extension) and lower limbs (hip flexion, knee extension, dorsiflexion of the foot) on both sides [21]. Among the 51 CSF samples from the 47 remaining patients, three samples were excluded due to the contamination of the peripheral blood; thus 48 CSF samples from 44 patients were used to analyze CSF ATP levels. In four patients, we collected CSF samples twice at different times, with either a two, three, six, or nine month interval, as it was assumed that the CSF ATP levels had changed with disease progression (Fig. 1). Ten CSF samples from patients with idiopathic normal pressure hydrocephalus (iNPH) were used as controls. CSF samples were obtained from lumbar puncture and stored at -80 °C until measurement. CSF samples were measured immediately after thawing, because of the limited stability of ATP in CSF.

### Measurement of the extracellular ATP levels and other biomarkers

A highly sensitive and automated ATP measurement device was used to measure extracellular ATP levels [22]. We selected the Luciferin-luciferase reagent HS attached to the Lucifell HS Set (Kikkoman Biochemifa Co., Ltd., Tokyo, Japan) as a luminescent reagent and diluted it tenfold with distilled water. The CSF specimens were diluted 20-fold with distilled water prior to measurement in order to prevent the inhibitory activity of high concentrations of chloride ions on the luminescent reagent. Luminescence was measured using a luminometer for 10 s after the addition of 50 μL of luminescent reagent to 10 μL of diluted specimen. We quantified extracellular ATPs level as the average of relative light intensities (count per second). The measurement was performed three times for each sample. A calibration curve of luminescence and ATP concentration was obtained using a tenfold dilution series of standard ATP solution adjusted with a saline solution diluted tenfold with distilled water. The ATP concentration was calculated from the luminescence value obtained from the calibration curve [18]. To confirm the precision and detection limit of this assay, we measured ATP standard solution prepared using commercial ATP and CSF collected from patients with iNPH as a diluent. These results indicated that the measurement error was up to 2.4%, and the ATP quantification limit in CSF was at least 2.0 × 10^–11^ mol/L.

We used the method using uricase and peroxidase for quantification of serum uric acid levels. Serum creatinine levels were determined by enzymatic method using creatinase, sarcosine oxidase and peroxidase.

### Statistical analysis

Statistical tests were performed using the JMP^Ⓡ^ Pro software 2019 ver 15.0 (SAS Institute Inc., Cary, NC, USA). A Weltch’s t test with holm correction and Wilcoxon test was used for mean comparison between groups. A Fisher’s exact test was used to compare the differences in frequency distribution on gender. Spearman’s test adjusted for age and sex was used to examine the correlation between variables. Results were considered significant when the *p*-values were < 0.05.

## Results

**Fig. 2:**
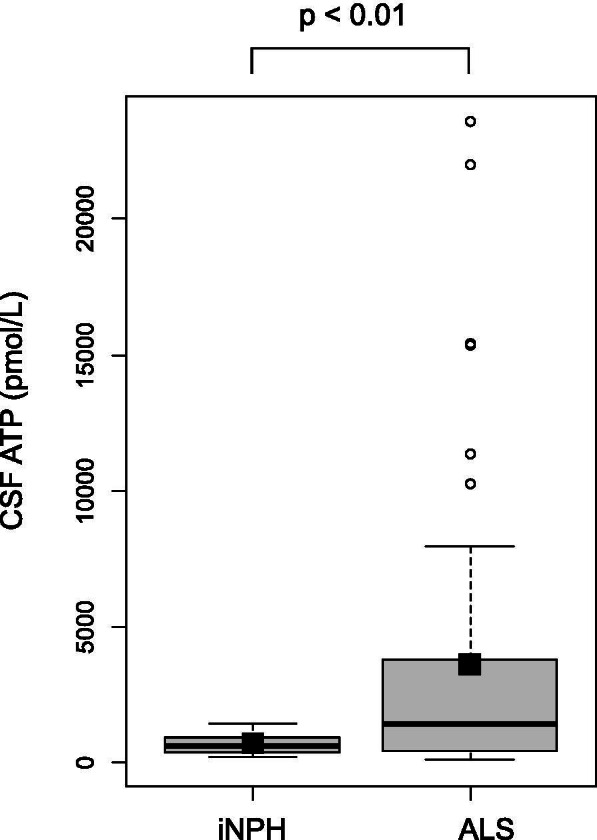
The boxplot of CSF ATP in iNPH and ALS patients. Closed squares and horizontal bars represent group means and group medians respectively. The mean ± SD of CSF ATP levels was 716 ± 411 vs. 3635 ± 5465 pmol/L in iNPH and ALS patients respectively. Weltch’s T test was used for comparing two groups. Value of *p *< 0.05 were statistically significant

**Fig. 3:**
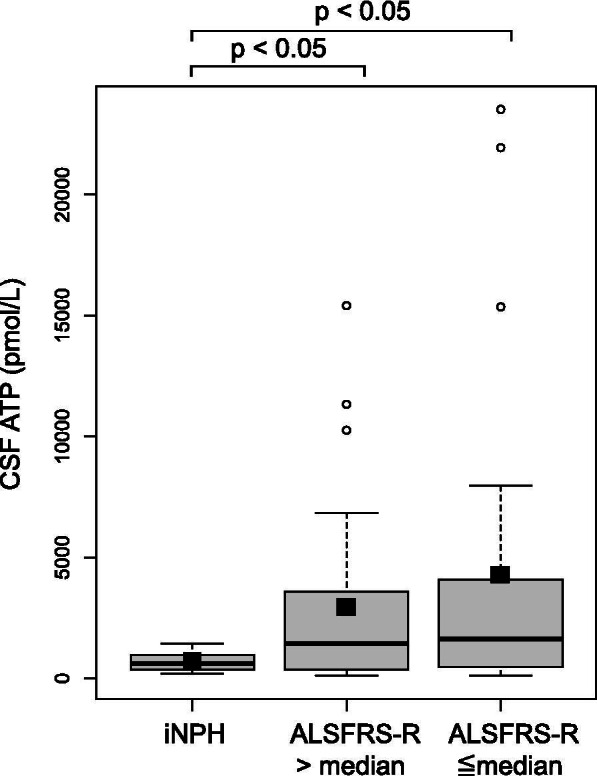
The boxplot of CSF ATP in iNPH and ALS patients (ALFFRS-R > median or ≦ median). Closed squares and horizontal bars represent group means and group medians respectively. The mean ± SD of CSF ATP levels was 716 ± 411 vs. 3379 ± 4300 vs. 3817 ± 6236 pmol/L in iNPH, ALSFRS-R > 42 (median value) and ALSFRS-R ≦ 42 group respectively. Weltch’s T test with Holm correction was used for comparing three groups. Value of *p *< 0.05 were statistically significant

**Fig. 4:**
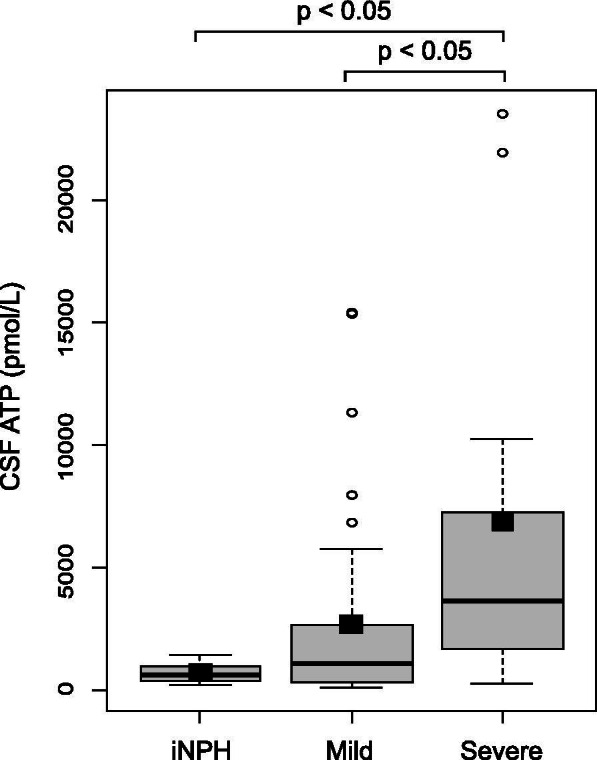
The boxplot of CSF ATP in iNPH, mild group and severe group of ALS patients. Closed squares and horizontal bars represent group means and group medians respectively. The mean ± SD of CSF ATP levels was 716 ± 411 vs. 2676 ± 3959 vs. 6860 ± 8312 pmol/L in iNPH, mild and severe groups respectively. Weltch’s T test with Holm correction was used for comparing three groups. Value of *p *< 0.05 were statistically significant

**Fig. 5:**
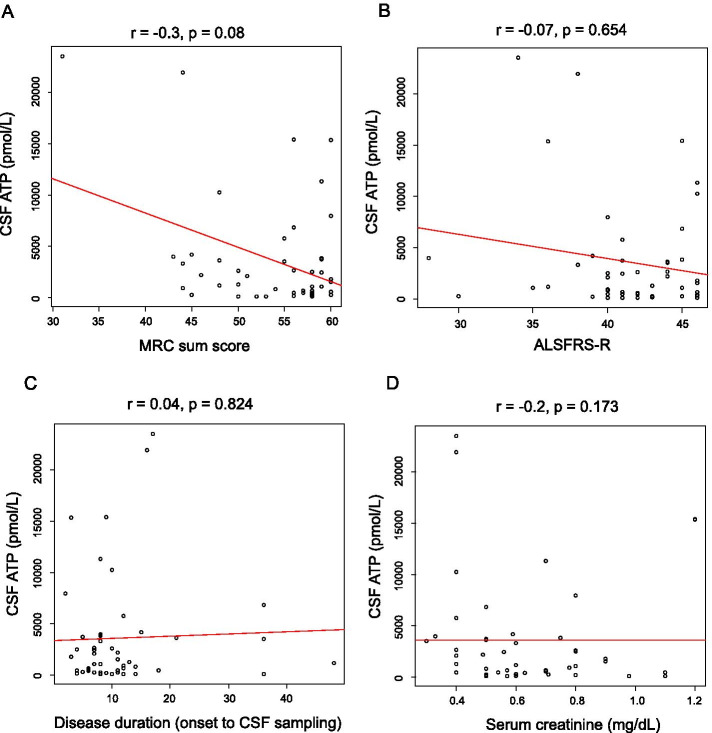
Correlation between CSF ATP levels and MRC sum score (**A**), ALSFRS-R (**B**), disease duration (onset to CSF sampling) (**C**) and serum creatinine (**D**). Spearman’s test adjusted for age and sex was used to examine the correlation between variables. r = correlation (95% CI). Value of *p *< 0.05 were statistically significant

CSF ATP levels were significantly higher in patients with ALS than in those with iNPH (716 ± 411 vs. 3635 ± 5465 pmol/L, *p *< 0.01) (Fig. 2). Patients with ALS were divided into two groups depending on the median value of the ALSFRS-R, and CSF ATP levels were compared between these groups. CSF ATP levels were significantly higher in both ALS groups than those in the iNPH group (716 ± 411 vs. 3379 ± 4300 pmol/L, 716 ± 411 vs. 3817 ± 6236, *p *< 0.05), but there were no significant differences in CSF ATP levels between the ALS groups (Fig. 3). Then the patients with ALS were divided into two groups depending on their disease severity evaluated using the MRC sum score. The mild group consisted of patients whose MRC sum scores were > 49, and the severe group consisted of those whose scores were ≤ 48. There were no significant differences in age, sex, and body mass index (BMI) between the two groups. ALSFRS-R was significantly lower in the severe group than in the mild group (37.9 ± 5.7 vs. 42.4 ± 2.8, *p *< 0.01). Disease duration from onset to CSF sampling was significantly longer in the severe group than in the mild group (15.8 ± 11.5 vs. 10.5 ± 8.4 months). Disease duration from onset to death or using a ventilator, and residual time after CSF sampling was shorter in the severe group than in the mild group (11.6 ± 5.5 vs. 18.2 ± 15.3 months) respectively, although without significant difference (Table 1). CSF ATP levels were significantly higher in the more severe group than in the iNPH group (6860 ± 8312 vs. 716 ± 411 pmol/L, *p *< 0.05) and mild group (6860 ± 8312 vs. 2676 ± 3959 pmol/L, *p *< 0.05) respectively, whereas there were no significant differences in the number of CSF cells and protein levels among the three groups (Table 2 and Fig. 4). Regarding serum molecules, serum creatinine levels were significantly lower in the severe group than in the mild group (0.51 ± 0.13 vs. 0.68 ± 0.23 mg/dL, *p *< 0.05), whereas uric acid levels were not significantly different (Table 2). In addition, there was a negative correlation between CSF ATP levels and MRC sum scores in patients with ALS upon a Spearman’s test adjusted for age and sex, although it was not significant (r = -0.3, *p *= 0.086) (Fig. 5A). There were no correlations between CSF ATP and ALSFRS-R, disease duration (onset to CSF sampling) or serum creatinine levels (Fig. 5B-D). Other factors such as BMI, disease duration (onset to death or ventilator), residual time, CSF cells, CSF protein levels, and serum uric acid levels did not show significant correlations with CSF ATP levels (data not shown).

**Table 1:** Demographic of patients with iNPH and ALS

	iNPH (*n *= 10)	Mild group (*n *= 37)	Severe group (*n *= 11)	*P* value
Age	71.1 ± 3.8	63.0 ± 13.3	69.5 ± 8.8	0.09
Male (%)	5 (50.0)	25 (67.6)	7 (63.6)	0.62
MRC sum score	59.3 ± 1.3	56.7 ± 3.0	44.2 ± 4.7	< 0.0001 *
BMI	23.4 ± 2.4	21.2 ± 3.0	21.1 ± 2.6	0.10
ALSFRS-R		42.4 ± 2.8	37.9 ± 5.7	< 0.01 *
Disease duration (onset to CSF sampling, months)		10.5 ± 8.4	15.8 ± 11.5	0.016 *
Disease duration ^a^ (onset to death or ventilator, months)		28.9 ± 18.0	24.3 ± 9.0	0.971
Residual time (months) ^a^		18.2 ± 15.3	11.6 ± 5.5	0.309

Data are presented as mean ± SD or number (%), * indicates *p *< 0.05 (wilcoxon test)

Mild group; MRC sum scores > 49, Severe group MRC sum scores ≤ 48

Residual time; duration to use of a ventilator or death after CSF sampling

*n *= number of samples, *ALSFRS-R* ALS functional rating scale-revised, *iNPH* Idiopathic normal pressure hydrocephalus, *BMI* Body mass index,

^a^ When comparing disease duration (onset to death or ventilator) or residual time, the mild group included 33 samples and severe group included 7 samples because remaining patients were alive without needing a ventilator

**Table 2:** Laboratory index of patients with iNPH and ALS

	iNPH (*n *= 10)	Mild group (*n *= 37)	Severe group (*n *= 11)	*P* value
CSF				
ATP (pmol/L)	716 ± 411	2676 ± 3959	6860 ± 8312	0.017 *
Cell (/μL)	0.6 ± 0.7	0.7 ± 0.8	0.6 ± 0.8	0.89
Protein (mg/dL)	38.2 ± 15.1	45.9 ± 18.8	42.5 ± 22.1	0.36
Serum				
Creatinine (mg/dL)	0.78 ± 0.17	0.68 ± 0.23	0.51 ± 0.13	< 0.01 *
Uric acid (mg/dL)	5.4 ± 1.2	5.0 ± 1.5	4.8 ± 1.0	0.546

Data are presented as mean ± SD, * indicates *p *< 0.05 (Wilcoxon test)

*n *= number of samples, *ATP* Adenosine triphosphate

## Discussion

In this report, we demonstrated that CSF ATP levels were significantly higher in patients with ALS than in those with iNPH. We also showed that CSF ATP levels were higher in patients with ALS who had longer disease durations and more severe symptoms, and the levels were inversely correlated with the MRC sum score. To our knowledge, this is the first report to evaluate CSF ATP levels in patients with ALS.

In the central nervous system, extracellular ATP regulates several physiological processes such as cell proliferation, differentiation, and apoptosis [23]. ATP is released by damaged neurons and is also present in the neuronal synaptic or secretory vesicles and is co-released to the extracellular space with neurotransmitters such as γ-aminobutyric acid (GABA) and glutamate [24]. ATP is released from not only neurons but also microglia and astrocytes through lysosome exocytosis [25]. The released ATP is degraded by ectonucleotidase, and an ATP metabolite binds to P2 purinergic receptors localized on the neuronal pre/post synaptic membranes, astrocytes, and microglia, which then participates in neuron-glial and inter-glial cell signaling [26, 27]. Activation of the astrocytes, derived from an ALS rat model, by stimulating the ATP receptor resulted in co-cultured motor neuron death, and this was prevented by the administration of an ATP receptor antagonist [28]. Thus, it has been assumed that ALS pathogenesis is partially attributed to the disability of neuron-glial cell interaction, that is, increased extracellular ATP released by damaged neurons is recognized as a damage-associated molecular pattern (DAMP) signal that activates the microglia and astrocytes, accompanied by the production of proinflammatory cytokines and excessive neuroinflammation and neuronal loss [29, 30]. Therefore, it is reasonable that CSF ATP levels are elevated in patients with ALS with progressive motor neuron degeneration in the central nervous system. We assayed CSF samples from patients with iNPH as controls. We could not use CSF samples from healthy people as a control, because it is difficult to ethically collect CSF samples from healthy people. Since CSF ATP levels increased with CSF pleocytosis and central nervous tissue damage, it is considered appropriate to use CSF samples from iNPH patients as a control, as they had no CSF pleocytosis or central nervous tissue injury. Indeed, in patients with iNPH, the levels of CSF neurofilament light chain (NfL), known as a marker of neuronal damage, was not significantly different from those in healthy control [31].

We showed that CSF ATP levels were higher in patients with ALS with advanced muscle weakness and were inversely correlated with the MRC sum score. ALSFRS-R and serum creatinine levels were significantly lower in the severe group than in the mild group. These results were consistent with those of a previous study, suggesting that plasma creatinine level correlates with ALS functional rating scale-revised ALSFRS-R and percent forced vital capacity (FVC), and significantly predicted survival [32].

It seems likely that a high concentration of extracellular ATP may induce motor neuron death and muscle weakness in patients with ALS. CSF ATP levels are elevated in patients with radiation-induced brain injury, and increased ATP exacerbates neuronal injury via the activation of microglia [33]. The mean CSF ATP level in those patients was approximately 10,000 pmol/L, which is not very different from that reported in patients with severe ALS in our study (3635 ± 5465 pmol/L). Thus, it is possible that ATP is released from damaged neurons, and this increase in ATP accelerates neuronal damage, although the exact mechanism behind this increase in the ATP levels remains unclear.

Serum NfL levels were elevated as a result of motor neuron degeneration in patients with ALS and were associated with prognosis [34]. Lower serum creatinine levels, as a result of muscle atrophy, are associated with shorter survival in patients with ALS [5]. Serum uric acid levels were elevated in patients with ALS treated with edaravone, reflecting the increased antioxidant capacity, and were associated with prognosis [35, 36]. In this study, CSF ATP levels showed very high variation among individuals. Due to these variation, CSF ATP levels may be less useful as a prognostic marker in patients with ALS. Conversely, in light of their potential as a therapeutic target of ATP receptor antagonists for patients with ALS [37], it is assumed that CSF ATP levels are useful biomarkers for evaluating the effect of such a new therapy, as would be the case for serum uric acid in the future.

This study has some limitations. First, this was a hospital-based cross-sectional study, and the sample size was relatively small. Further study is needed to clarify the relationship between CSF ATP levels and several inflammatory cytokines such as TNF-α, IL-6, and COX-2 as well as between CSF ATP levels and blood or CSF NfL, which reflect motor neuron degeneration in patients with ALS. Second, we have not evaluated CSF ATP levels of patients with other neurological diseases like.

Alzheimer’s disease, Parkinson’s disease, multiple sclerosis. It is necessary to elucidate whether increase of CSF ATP level is specific for ALS in further studies.

## Conclusions

In summary, we demonstrated a relationship between CSF ATP levels and MRC sum score and serum creatinine levels in patients with ALS. CSF ATP levels may be a useful biomarker for evaluating disease severity in patients with ALS. Further studies should be conducted to confirm our results.

## Data Availability

The datasets used and analyzed during this study are available from the corresponding author on reasonable request.
